# How do pandemic changed obstetric and neonatal outcomes: experience from a tertiary care center

**DOI:** 10.1590/1806-9282.20250553

**Published:** 2025-10-17

**Authors:** Samet Kırat

**Affiliations:** 1 Kafkas University, Faculty of Medicine, Department of Gynecology and Obstetrics – Kars, Turkey.

**Keywords:** COVID-19, Comorbidity, Maternal health, Neonatal intensive care unit

## Abstract

**OBJECTIVE:**

The coronavirus disease pandemic has significantly impacted maternal and neonatal health outcomes.

**METHODS:**

This retrospective study compared the health outcomes of 1,556 pregnant women and their newborns who gave birth before and during the pandemic at a tertiary university hospital. Demographic and clinical data were collected from hospital medical records. The primary outcome was the association between coronavirus disease and neonatal intensive care unit admission rates, while the secondary outcomes included birth weight, gestational age at delivery, 5th-min APGAR scores, and maternal complications.

**RESULTS:**

During the pandemic, screening test rates (58.9 vs. 33.2%, p<0.001), gestational diabetes mellitus prevalence (9.8 vs. 4.2%, p<0.001), and neonatal intensive care unit admission rates (8 vs. 4.4%, p=0.003) were significantly higher than those in the pre-pandemic period. Preterm birth rates were lower during the pandemic (12.5 vs. 25.1%, p<0.001). Logistic regression analysis revealed that infants born during the pandemic had a significantly higher likelihood of neonatal intensive care unit admission (OR 1.910, 95%CI 1.237–2.949, p=0.004).

**CONCLUSION:**

These findings highlight the need for health systems to be prepared and resilient to public health crises, ensuring uninterrupted health services for pregnant women and newborns.

## INTRODUCTION

The COVID-19 pandemic has emerged as a global crisis that has significantly affected health systems and individual health outcomes. This process has also brought various challenges for pregnant and laboring mothers and their newborn babies. Understanding the differences between mothers who gave birth before and during the pandemic is important for health policies and delivery services. In this context, comparing maternal and neonatal health outcomes is a critical step in assessing the adequacy of existing health services^1^.

Increased stress, anxiety, and changes in access to healthcare services in mothers giving birth during the pandemic have directly affected the pregnancy process. Research shows that mothers have experienced higher rates of complications such as preeclampsia, gestational diabetes, and hypertension during the pandemic^2^. Additionally, disruptions in routine prenatal follow-up and delays in medical interventions may have further exacerbated these complications. It has also been reported that cesarean delivery rates are increasing, and the risk of preterm birth is rising in some countries, potentially due to concerns about maternal and fetal well-being amid pandemic-related uncertainties^3^.

In terms of newborn babies, low birth weight and neonatal intensive care unit (NICU) admission rates increased in babies born during the pandemic. Babies born during the pandemic period are more likely to show developmental delays in the first 3 months^4^. These findings highlight the importance of uninterrupted prenatal and postnatal care in ensuring optimal neonatal outcomes. However, some studies have found that restrictions on access to prenatal healthcare have adverse effects on fetal development, emphasizing the need for alternative healthcare strategies in times of crisis^5^.

This study aims to evaluate the effects of the COVID-19 pandemic on maternal complications and neonatal health, and to compare the health outcomes of women who gave birth before and during the pandemic and newborns. Restrictions in access to healthcare services during the pandemic, increased psychosocial stress, and the reflections of changing clinical practices on maternal and infant health were examined from a holistic perspective. The limited number of comparative and center-based analyses in the existing literature reveals the unique contribution of this study; the findings obtained are expected to provide guidance for centers providing services under similar conditions.

## METHODS

### Patients and data collection

**Figure 1:**
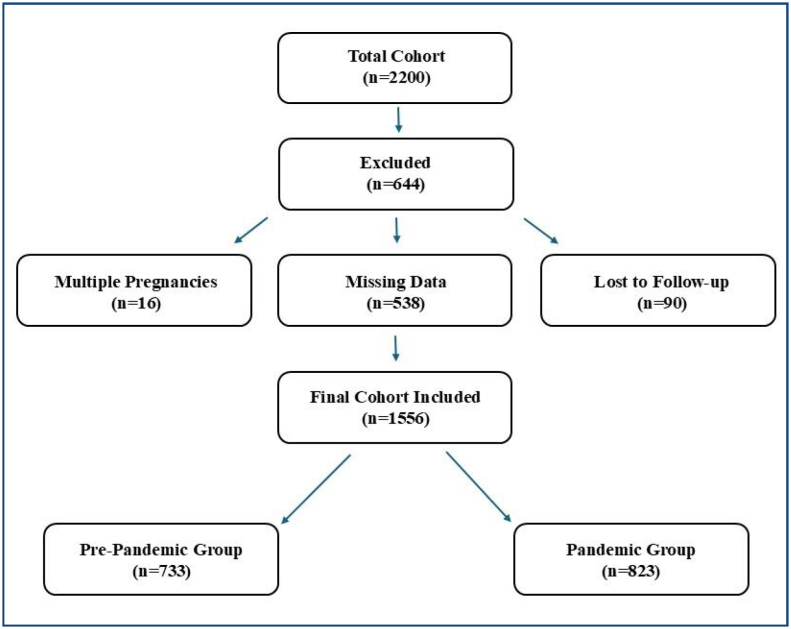
CONSORT flow diagram showing patient selection and stratification by pre-pandemic and pandemic periods.

Between January 1, 2018 and December 31, 2023, a total of 1,556 pregnant women who were admitted to the Obstetrics and Gynecology Clinic of a tertiary university hospital were included in this retrospective study. Demographic and clinical data, including age, gravida, parity, comorbidities, smoking status, screening test results, year of birth, gestational age, infant birth weight, 5th-min APGAR scores, NICU hospitalization history, and NICU admission diagnosis, were obtained from hospital medical records. Pregnant women who visited the Department of Obstetrics and Gynecology within the specified period, but had missing data in their medical records, were excluded from the study. The first confirmed case of COVID-19 in Turkey was officially announced by the Ministry of Health on March 11, 2020, which marked the beginning of the pandemic. This date was used as a reference point for pre-pandemic (January 1, 2018–March 10, 2020) and pandemic (March 11, 2020–December 31, 2023) period comparisons^6^. A CONSORT flow diagram (Figure 1) illustrates the patient selection process, detailing the number of patients included and excluded at each stage, as well as stratification by pre-pandemic and pandemic periods.

### Comorbidities

Maternal comorbidities were defined as pre-existing medical conditions diagnosed before or independent of pregnancy-related changes. These included obesity (pre-pregnancy BMI ≥30 kg/m^2^), thyroid disorders, epilepsy, thrombophilia, rheumatoid arthritis, immune thrombocytopenic purpura, asthma, bipolar mood disorder, bronchiectasis, celiac disease, and polycystic ovary syndrome. Diagnoses were confirmed using ICD-10 codes, test results, and clinical evaluations from hospital records. In contrast, pregnancy-related conditions such as gestational diabetes mellitus (GDM), preeclampsia, and gestational hypertensive disorders are classified as pregnancy outcomes and analyzed separately in the results. A comprehensive list of comorbidities and definitions is in Table 1.

**Table 1:** Demographic data of the total cohort.

		Total cohort (n=1,556)
Age (years) [median (min–max)]		33 (19–50)
Smoking (n, %)		72 (4.6%)
Parity (n, %)		
	Nullipar	877 (56.4%)
	Multipar	679 (43.6%)
Comorbidity (n, %)		78 (5%)
	Hypothyroidism	42 (2.7%)
	Epilepsy	7 (0.4%)
	Thrombophilia	7 (0.4%)
	Rheumatoid arthritis	2 (0.1%)
	Immune thrombocytopenic purpura	2 (0.1%)
	Asthma	1 (0.1%)
	Bipolar mood disorder	1 (0.1%)
	Bronchiectasis	1 (0.1%)
	Celiac disease	1 (0.1%)
	Hyperthyroidism	1 (0.1%)
	Polycystic ovary syndrome	1 (0.1%)
Pregnancy-related conditions (n, %)		112 (7.2%)
	Gestational diabetes mellitus	101 (6.5%)
	Gestational hypertensive disorders	10 (0.6%)
	Preeclampsia	2 (0.1%)
Screening tests (n, %)		728 (46.8%)
	Dual test	569 (36.6%)
	Triple test	65 (4.2%)
	OGTT	269 (17.3%)
	Detailed USG	121 (7.8%)
Year of birth (n, %)		
	2018	31 (2%)
	2019	601 (38.6%)
	2020	298 (19.2%)
	2021	113 (7.3%)
	2022	239 (15.4%)
	2023	274 (17.6%)
Period (n, %)		
	Pre-pandemic period	733 (47.1%)
	Pandemic period	823 (52.9%)
Birth week (n, %)		
	<37 weeks	287 (18.4%)
	37–42 weeks	1,266 (81.4%)
	>42 weeks	3 (0.2%)
Infant birth weight (n, %)		
	<2,500 g	105 (6.7%)
	2,500–4,000 g	1,424 (91.5%)
	>4,000 g	27 (1.7%)
5th-min APGAR score (median (min–max))		9 (0-10)
NICU (n, %)		98 (6.3%)
	TTN	50 (3.2%)
	Bacterial sepsis	37 (2.4%)
	RDS	8 (0.5%)
	MAS	5 (0.3%)

OGTT: Oral Glucose Tolerance Test, USG: ultrasonography, NICU: Neonatal Intensive Care Unit, TTN: transient tachypnea of the newborn, MAS: meconium aspiration syndrome, RDS: respiratory distress syndrome.

### Primary and secondary outcomes

The primary outcome of this study was the association between COVID-19 and NICU hospitalization rates. The secondary outcomes included birth weight, gestational age at delivery, 5th-min APGAR scores, and maternal complications (GDM, preeclampsia, and gestational hypertensive disorders).

### Statistical analysis

Statistical analyses were performed using SPSS (version 24.0; IBM Corp., Armonk, NY, USA) and GraphPad Prism version 8 (GraphPad Software, San Diego, CA, USA). Categorical variables were presented as numbers with percentages and assessed using chi-square or Fisher's exact test. Continuous variables are expressed as median (minimum–maximum) or mean±standard deviation (SD) based on Kolmogorov-Smirnov normality test results. Group comparisons used Student's t-test (for normally distributed data) or Mann-Whitney U test (for non-normally distributed data). Logistic regression analysis assessed the association between COVID-19 infection and NICU admission rates, adjusting for maternal age, comorbidities, gestational age at delivery, smoking status, and parity. Odds ratios (ORs) with 95%CIs were reported. Statistical significance was set at p<0.05.

### Ethics committee approval

This study was approved by the Non-Interventional Clinical Research Ethics Committee of the Kafkas University Faculty of Medicine (Approval Date: 27/02/2024, Decision No 80576354-050-99/388) and conducted in accordance with the ethical principles of the Declaration of Helsinki for human biomedical research.

## RESULTS

### Demographic data of the total cohort

A total of 1,556 pregnant women were included in the study. The median age of the participants was 33 (19–50) years, 56.4% were nulliparous, and 43.6% were multiparous. Smoking was observed in 4.6% (n=72) of patients. Comorbidities were present in 5% (n=78) of patients, and the most common disease was hypothyroidism (2.7%). Pregnancy-related conditions were detected in 7.2% (n=112) of patients, and the most common condition was GDM (6.5%). Screening tests were performed in 46.8% (n=728) of the pregnant women, and the most common test was the dual test (36.6%). When birth outcomes were analyzed, 47.1% (n=733) of births occurred before the COVID-19 pandemic and 52.9% (n=823) occurred during the pandemic period. The rate of preterm birth was 18.4% (n=287), while 6.3% (n=98) were admitted to the NICU. The most common reasons for hospitalization were transient tachypnea (3.2%) and bacterial sepsis (2.4%). The median 5-min APGAR score was 9 (0–10). Detailed data are presented in Table 1.

### Comparison of pre-pandemic and pandemic period pregnant women

A total of 733 deliveries occurred before the COVID-19 pandemic, whereas 823 occurred during the pandemic period. Pregnant women in the pre-pandemic group were significantly older (34 vs. 32 years, p<0.001) and had a higher nulliparity rate (63.6 vs. 49.9%, p<0.001), whereas multiparity was more common during the pandemic (50.1 vs. 36.4%, p<0.001). Pregnancy-related conditions, particularly GDM, were significantly more common during the pandemic (9.8 vs. 4.2%, p<0.001). The screening rate was also notably higher in the pandemic group (58.9 vs. 33.2%, p<0.001). Preterm birth rates were lower during the pandemic period than during the pre-pandemic period (12.5 vs. 25.1%, p<0.001). The NICU hospitalization rate was significantly higher during the pandemic period than during the pre-pandemic period (8 vs. 4.4%, p=0.003), mainly due to increased cases of sepsis (3.6 vs. 1%, p=0.001). The median 5th-min APGAR score was slightly lower during the pre-pandemic period (9 [0–10] vs. 9 [5–10], p=0.002). Logistic regression analysis revealed that the likelihood of NICU hospitalization of infants born during the pandemic period increased significantly compared with that in the pre-pandemic period (OR 1.910, 95%CI 1.237–2.949, p=0.004). Detailed data are presented in Table 2.

**Table 2:** Comparison of pre-pandemic and pandemic period.

		Pre-pandemic period (n=733)	Pandemic period (n=823)	p-value
Age (years) [median (min–max)]		34 (20–50)	32 (19–50)	**<0.001**
Smoking (n, %)		29 (4%)	43 (5.2%)	0.234
Parity (n, %)				
	Nullipar	466 (63.6%)	411 (49.9%)	**<0.001**
	Multipar	267 (36.4%)	412 (50.1%)	**<0.001**
Comorbidity (n, %)		39 (5.3%)	39 (4.7%)	0.600
	Asthma	1 (0.1%)	0 (0%)	0.289
	Bipolar mood disorder	1 (0.1%)	0 (0%)	0.289
	Bronchiectasis	0 (0%)	1 (0.1%)	0.345
	Celiac disease	0 (0%)	1 (0.1%)	0.345
	Epilepsy	2 (0.3%)	5 (0.6%)	0.458
	Hypothyroidism	21 (2.9%)	21 (2.6%)	0.823
	Hyperthyroidism	0 (0%)	1 (0.1%)	0.345
	Polycystic ovary syndrome	1 (0.1%)	0 (0%)	0.289
	Rheumatoid arthritis	2 (0.3%)	0 (0%)	0.134
	Immune thrombocytopenic purpura	0 (0%)	2 (0.2%)	0.182
	Thrombophilia	2 (0.3%)	5 (0.6%)	0.458
Pregnancy-related conditions (n, %)		31 (4.2%)	81 (9.8%)	**<0.001**
	Gestational diabetes mellitus	24 (3.3%)	77 (9.4%)	**<0.001**
	Gestational hypertensive disorders	7 (1%)	3 (0.4%)	0.205
	Preeclampsia	0 (0%)	2 (0.2%)	0.182
Screening tests (n, %)		243 (33.2%)	485 (58.9%)	**<0.001**
	Dual test	152 (20.7%)	417 (50.7%)	**<0.001**
	Triple test	19 (2.6%)	46 (5.6%)	**0.003**
	OGTT	95 (13%)	174 (21.1%)	**<0.001**
	Detailed USG	33 (4.5%)	88 (10.7%)	**<0.001**
Birth week (n, %)				
	<37 weeks	184 (25.1%)	103 (12.5%)	**<0.001**
	37–42 weeks	547 (74.6%)	719 (87.4%)	**<0.001**
	>42 weeks	2 (0.3%)	1 (0.1%)	0.604
Infant birth weight (n, %)				
	<2,500 g	56 (7.6%)	49 (6%)	0.186
	2,500–4,000 g	663 (90.5%)	761 (92.5%)	0.154
	>4,000 g	14 (1.9%)	13 (1.6%)	0.761
5th-min APGAR score [median (min–max)]		9 (0–10)	9 (5–10)	**0.002**
NICU (n, %)		32 (4.4%)	66 (8%)	**0.003**
	TTN	23 (3.1%)	27 (3.3%)	0.988
	Sepsis	7 (1%)	30 (3.6%)	**0.001**
	MAS	0 (0%)	5 (0.6%)	0.064
	RDS	4 (0.5%)	4 (0.5%)	1.0

OGTT: Oral Glucose Tolerance Test, USG: ultrasonography, NICU: Neonatal Intensive Care Unit, TTN: transient tachypnea of the newborn, MAS: meconium aspiration syndrome, RDS: respiratory distress syndrome. The bold values indicate statistically significant results (p<0.05).

## DISCUSSION

In this study, we compared pregnant women who gave birth during and before the pandemic with newborns born to these women to assess the effects of COVID-19 on maternal and neonatal outcomes. Our findings revealed that rates of screening tests (p<0.001), GDM prevalence (p<0.001), and NICU admission (p<0.001) were significantly higher during the pandemic period than pre-pandemic. In contrast, preterm birth rates were higher in the pre-pandemic period (p<0.001).

During the pandemic, screening tests performed during pregnancy decreased significantly due to difficulties accessing health services and fear of contamination. In some countries, a decrease of up to 30% in routine pregnancy follow-up, ultrasound, and other tests has been reported^7^. However, this decline gradually decreased as the pandemic progressed, and interest in screening tests increased, particularly in high-risk pregnant women^8^. Screening tests were performed more regularly in pregnant women with comorbidities, and some participated more frequently with the spread of telemedicine services^1,9,10^. In our study, the rate of screening tests during the pandemic was significantly higher than before the pandemic (p<0.001). This may be due to increased perception of high risk and stricter follow-up in pregnant women with comorbidities.

Decreased physical activity, unhealthy lifestyle changes, and reduced healthcare access during the pandemic have increased comorbidities such as obesity, diabetes, hypertension, and preeclampsia in pregnant women^7,8^. The inflammatory effects of COVID-19, increased stress, and healthcare system overload have contributed to the spread of these comorbidities^1,9-12^. Studies have reported that comorbidity rates, which were between 18 and 25% before the pandemic, increased to 30–41% during the pandemic^11-13^. In our study, a notable increase was found in GDM rates, with pregnant women with GDM increasing three times compared to the pre-pandemic period (9.4 vs. 3.3%, p<0.001). Stress caused by the pandemic and decreased physical activity due to quarantine measures may be reasons for this increase.

Fetal oxygenation and nutrition may be impaired due to COVID-19 effects on the placenta, including inflammation, vascular damage, and platelet–fibrin deposition, potentially increasing the risk of intrauterine growth retardation (IUGR), low birth weight (LBW), and preterm delivery^14,15^. Pre- and post-pandemic preterm birth rates decreased from 9.4 to 6.8% in Spain^1^ but increased from 10.2 to 11.5% in the USA^16^ and from 10.1 to 14.2% in Italy^17^. The preterm birth rate has increased from 6.3 to 9.1% in the UK and from 8.4 to 12% in Israel^1,18^. In our study, the preterm birth rate decreased from 25.1 to 12.5% (p<0.001), while it decreased from 7.6 to 6% (p=0.186). This may be related to stricter compliance with prenatal follow-ups and easier access to health services in our country.

NICU hospitalization rates increased significantly during the pandemic period compared to pre-pandemic. This increase is due to higher fetal distress rates from COVID-19 infection, premature births, and maternal comorbidities^10,19,20^. Difficulties accessing health services and disrupted prenatal care also contributed to higher fetal distress rates^19,20^. Previous studies showed NICU admission rates increased from 5–10% pre-pandemic to 9–18% during, associated with preterm delivery and maternal comorbidities^11,16,21^. Our study's increase in NICU hospitalization rate, from 4.4% pre-pandemic to 8% during (p=0.003), aligns with literature trends.

One limitation of this study is its regional and single-center focus, potentially limiting result generalizability. Health services and measures vary across regions during the pandemic, making results potentially specific to local conditions. Factors such as socioeconomic status and health literacy of pregnant women could not be fully controlled, possibly influencing COVID-19-related outcomes. The study's strengths include addressing effects on maternal and infant health during the pandemic and allowing comparisons with existing literature. It is based on a large dataset, examines important clinical outcomes such as NICU admission rates, and highlights increased NICU admissions in pregnant women with COVID-19.

## CONCLUSION

The COVID-19 pandemic has significantly affected maternal and neonatal health. Experience shows health systems need to be prepared and resilient to public health crises. Challenges accessing hospitals, increased NICU admission rates, and complications in pregnant women with COVID-19 highlight the importance of sustainability and capacity-building for health services. Future research should focus on strategies to increase health system preparedness and resilience, and examine long-term effects of the pandemic on maternal and neonatal health. Public health planning must ensure health services continue without disruption during future outbreaks.

## Data Availability

The datasets generated and/or analyzed during the current study are available from the corresponding author upon reasonable request.
